# Classical conditioned responses to absent tones

**DOI:** 10.1186/1471-2202-7-60

**Published:** 2006-08-17

**Authors:** Marc Bangert, Uwe Jürgens, Udo Häusler, Eckart Altenmüller

**Affiliations:** 1 Institute of Music Physiology and Musicians Medicine, Hanover University of Music and Drama, Hohenzollernstrasse 47, D-30161 Hanover, Germany; 2 Dept of Neurology, Beth Israel Deaconess Medical Center and Harvard Medical School, 330 Brookline Ave (Palmer 127), Boston, MA 02215, USA; 3 German Primate Center, Kellnerweg 4, D-37077 Göttingen, Germany

## Abstract

**Background:**

Recent evidence for a tight coupling of sensorimotor processes in trained musicians led to the question of whether this coupling extends to preattentively mediated reflexes; particularly, whether a classically conditioned response in one of the domains (auditory) is generalized to another (tactile/motor) on the basis of a prior association in a second-order Pavlovian paradigm. An eyeblink conditioning procedure was performed in 17 pianists, serving as a model for overlearned audiomotor integration, and 14 non-musicians. Results: During the training session, subjects were conditioned to respond to auditory stimuli (piano tones). During a subsequent testing session, when subjects performed keystrokes on a silent piano, pianists showed significantly higher blink rates than non-musicians.

**Conclusion:**

These findings suggest a tight coupling of the auditory and motor domains in musicians, pointing towards training-dependent mechanisms of strong cross-modal sensorimotor associations even on sub-cognitive processing levels.

## Background

Only few domains of skilled sensorimotor behavior involve fast integration of auditory and motor representations to a higher degree than the performance of instrumental music. Perhaps only speech surpasses this level of precise audiomotor co-representation. Speech, however, always bears the experimental disadvantage of virtually no access to an untrained control group.

In playing an instrument, the performance targets of the highly trained movement patterns are sequences of acoustic events. Therefore, any self-monitoring during musical performance has to rely on quick feedforward and feedback models that link the audible targets to the respective motor programs. Lifetime practice has been suggested and been shown even to alter macrostructural brain anatomy [1-3].

Recent studies on musicians have accumulated evidence for a tight connection between action and perception of the sensory feedback so that a mental representation of the feedback seems to be generated even if its physical presence is experimentally suppressed: Lotze *et al*. [4] contrasted fMRI scans of professional musicians and amateurs who tapped out an imagined and well-known piece of music. The professionals showed stronger activations of primary auditory cortex. Conversely, anticipatory movement and cross-modal representations seem to be involuntarily evoked when the highly trained subject is presented with the sensory target of the intended action. Bangert *et al*. [5] have shown professional pianists to co-activate motor and auditory areas in tasks requiring either pressing keys of a silenced keyboard, or listening to simple tone sequences. Non-pianists lacked this co-activation and showed distinct activational patterns of either motor or auditory areas, depending on the actual task. Bangert & Altenmüller [6] demonstrated that these cross-activation effects emerge in brain activity patterns after just one session of piano practice, indicating the relevance of such a mental corepresentation. The coupling of auditory and motor processing turned out to be rather strong, so that the experimental stimulation of one of those two partial representations alone produces an automatic co-activation of the other (on the topic of automaticity, see also [7-9]). The occurrence of this characteristic co-activity seemed to be independent of the degree of attention to the task. The circuits in question appear to be active even when the subject was not attending.

The present study is based on the described phenomenon that a professional pianist seems to have an obligatory covert auditory image when pressing a key of a muted piano. The behavioral paradigms employed in the previously cited studies, however, have not conclusively demonstrated to which degree the process is really pre-attentive and subconscious, or just obligatory yet based on an automatic conscious strategy. Post-hoc self-reports of whether or not the participants have been aware of the phenomenon might not be sufficient here; a paradigm that utilizes a distractor task with massive cognitive demand is desirable in order to put the genuine automatic nature of the process to the test.

A promising way to directly address this is to combine classical reflex conditioning (as reflexes functionally can not occur solely on a highest-order cognitive processing stage) with a highly demanding distractor task within a different domain.

The present study sets out to look at the behavioral effects associated with a very basic reaction – the eyeblink reflex to an airpuff – and whether it can be conditioned and evoked under circumstances where the sensory stimulus is physically absent and is only mediated through a putative sensorimotor coupling.

Pianists differ from non-pianists by having acquired an implicit knowledge of the 'tonotopic' organisation of key-pitch associations on a piano keyboard. This knowledge may be considered as a conditioned reflex in itself, with the tone as the unconditioned stimulus (US), the sensation of the tone as the unconditioned reaction (UR), the visual and tactile features of the keyboard as the conditioned stimuli (CS), and the knowledge (mental image) of the tone produced by a specific key as the conditioned reaction (CR). If this consideration is correct, one can go ahead and test for a conditioned reflex of second order in the Pavlovian sense, with the airpuff as the unconditioned stimulus, eyeblink as unconditioned and conditioned reaction, tone as the conditioned stimulus of first order, and the visuo-tactile features of the keyboard as the conditioned stimulus of second order. It is a general characteristic of second-order conditioned reflexes that the conditioned stimulus of second order inherits the CR eliciting properties of the first order conditioned stimulus simply on the basis of their mutual contingency [10]. Therefore, if a second-order effect is present in a tactile-motor task with a high degree of cross-modal contingency in the specific external context, this is strong evidence for the respective cross-modal contingency in brain areas involved in Pavlovian reflex conditioning.

In the conditioning procedure, a short air puff against the cornea is used as the unconditioned (aversive) stimulus, the closing of the eye represents the unconditioned reaction; a tone preceding the airpuff is used as the conditioned stimulus, and eye closure to the tone serves as the conditioned reaction. The question posed is the following: In musicians, does a key press on a muted piano still evoke the conditioned response to the associated – but inaudible – tone?

Two groups of subjects (14 non-musicians (NM) and 17 professional pianists (PP)) were tested in two different, but similarly designed sessions, and a baseline condition. All three sessions were 'masked' from the subjects' attention by a demanding distractor task (details see Methods section). In Session 0 (Baseline – prior to conditioning), random stimulus presentations out of a set of five piano tones were administered to evaluate the spontaneous eyeblink behaviour without a US. Session 1 (Auditory Conditioning) was the classical conditioning procedure. Subjects were acoustically stimulated with multiple randomized piano tone presentations (the five different tones c', d', e', f', g'). One of these tones was (consistently within each subject) assigned to be the conditioned stimulus (CS+, target) and therefore accompanied by a short air puff delivered to the eye. The unconditioned stimulus (US) was delivered only after the CS+ tone but not after any of the other tones (CS-, nontarget). Session 2 (Silent Tapping) required the subjects to voluntarily press down the five piano keys corresponding to the five notes, one at a time. The crucial manipulation was that the sound of the electronic piano was turned off, so that in this session neither the US nor the CS were present. In order to investigate the influence of processing time constraints, the CS-US latencies were varied in four subgroups to assume the values 200 ms, 400 ms, 800 ms, and 1000 ms.

The specific hypotheses were as follows:

• Principal Hypothesis: Following the conditioning procedure to a tone, silent keypresses will elicit an eyeblink response in pianists but not in non-musicians.

• Additional Hypothesis 1: The eyeblink elicited by silent key presses displays a specificity to the key associated with the target tone that was originally coupled with the US.

• Additional Hypothesis 2: The parameters relevant to detection, such as sensitivity and overall excitability of the CR, show a correlation with processing time constraints, i.e. with different CS-US delays.

## Results

### Group averages

**Figure 1:**
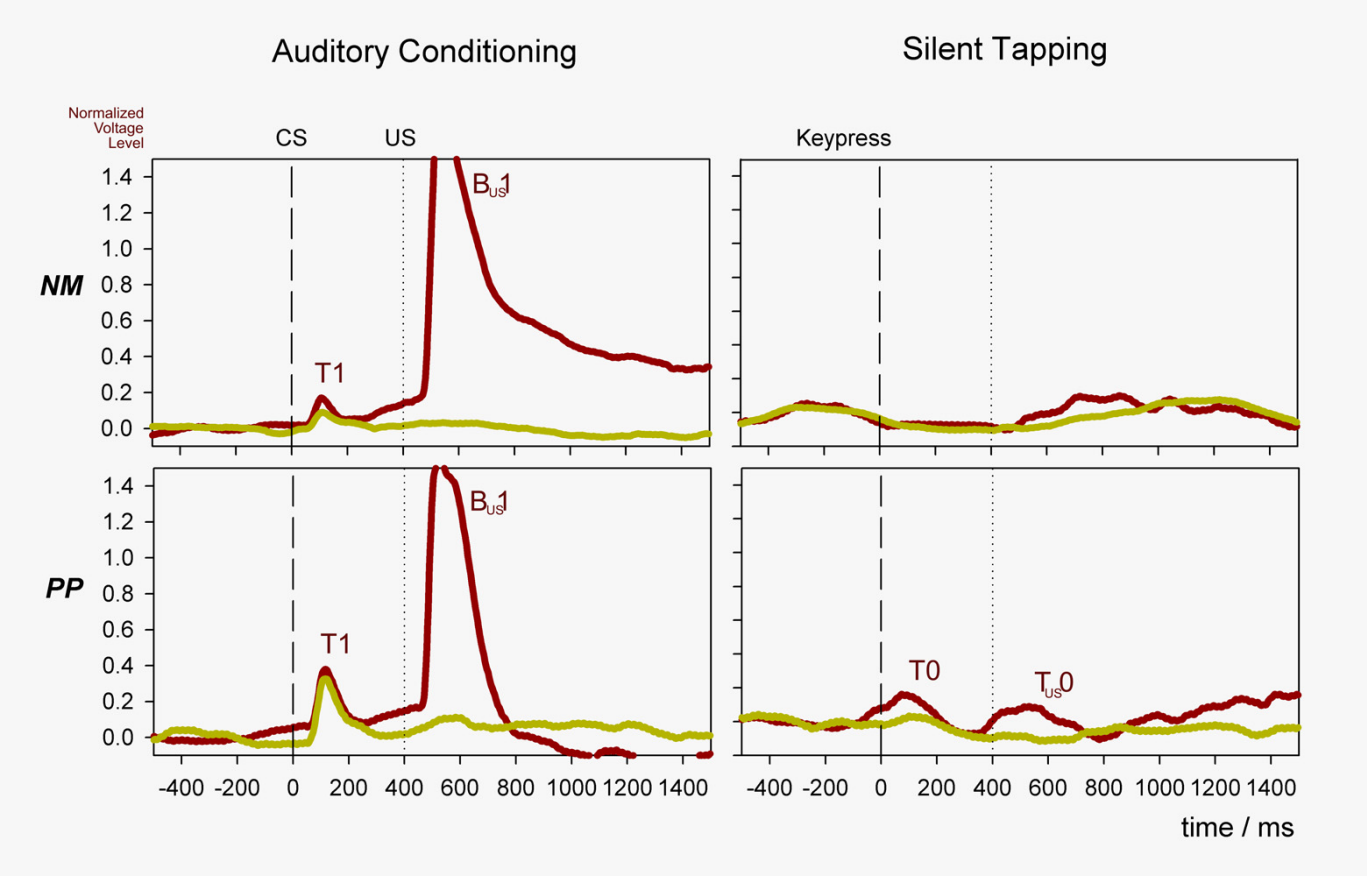
**Example of group-averaged eyeblink signal for the Auditory Conditioning session (left) and the Silent Tapping session (right)**. Example of a group-averaged time series of normalized event-related eyeblink signal for the Auditory Conditioning session (left) and the Silent Tapping session (right)(n = 9). Non-musicians (NM) are depicted in the upper and professional pianists (PP) in the lower panel. The red curve is the response to the target tone and the yellow curve is the response to nontarget tones. t = 0 (dashed line) refers to tone (CS) onset or keystroke, respectively. The dotted line marks the onset of the airpuff (US, at t = 400 ms), but note that only during the Auditory Conditioning the US was present. Peaks with a distance of more than standard deviation from baseline (SD curves not shown) have been labeled T (twitches) and B (blinks), followed by a number indicating the latency from event onset (e.g. "1" = 100 ms), or the relative latency from US onset (subscript US for unified nomenclature despite varying CS-US latencies). NB: (1) In the average, n(nontargets) = 4*n(target) applies. (2) In the PP Silent Tapping condition, peaks received the labels T0 and T_US_0 because the majority of the individual spikes coincided with the reference time (compare Fig.2), although the peak of the averaged timeseries appears at 100 ms offset.

Group averages of the normalized event-related epochs were calculated for each CS-US interval independently. Fig. 1 gives the results for the subject group with the CS-US interval 400 ms as an example. During the Auditory Conditioning session, the typical shape of a classically conditioned eyeblink can be seen: the eyelid closes compulsively shortly after the onset of the airpuff (B_US_0; labeling conventions see Fig 1 legend, and below), but also shows a movement shortly after the presentation of the tone as well as immediately before the UR in the form of a slowly ascending ramp (CR) (Fig. 1, left panels). In both target and nontarget stimulus presentations, the average signal shows a small peak at a latency of about 100 ms (T1). In both the NM and PP groups, the eyelid reaction curve immediately after the CS is identical for the target and nontarget tones, while the subsequent ramp is more pronounced in the target condition than in the nontarget condition. The build-up of a ramp preceding the US was used to determine whether the auditory conditioning procedure had been successful. (An additional, separate session to check for conditioning success, i.e. CS-only presentation, was not performed after pilot experiments had shown a relatively quick 'wash-out' curve of the CR, which thus may not be preserved into the Silent Tapping session.)

The Silent Tapping session revealed two small waves in the PP group absent in the NM group (Fig. 1). These waves appeared to be accompanying both the finger movement (keystroke) and the moment when an airpuff would have ensued during the Auditory Conditioning. These very small peaks appear to be more pronounced when the piano key assigned to the target tone was operated (red curve), although this difference was not significant.

### Peri-Stimulus Time Histograms (PSTH)

**Figure 2:**
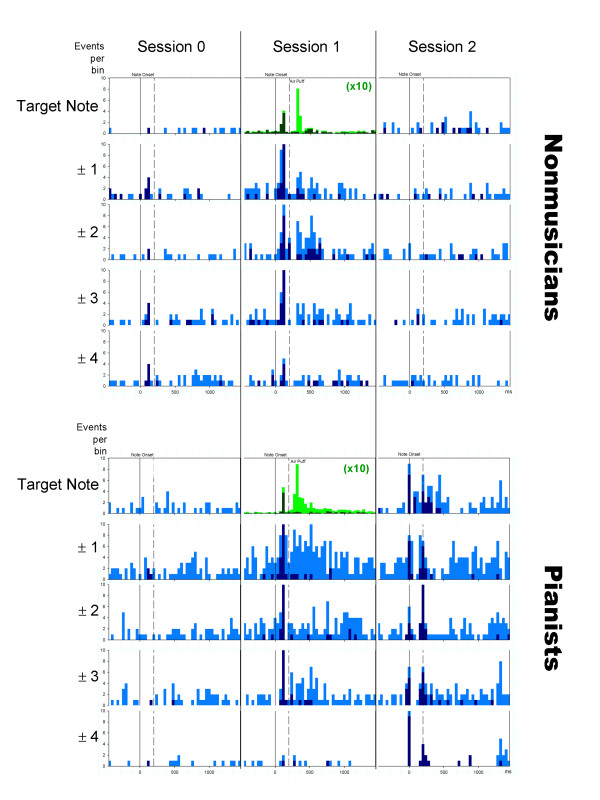
**Peri-Stimulus Time Histograms**. PSTHs of eye blinks (light green and light blue) and eyelid twitches (dark green and dark blue) during the three experimental sessions (n = 5). Baseline Session 0: left column; Auditory Conditioning Session 1: center column; Silent Tapping Session 2: right column. The histogram bins were 40 ms wide; bars are stacked. The only condition with the US (airpuff) actually present have been highlighted in green (Please note that the y-axis [events per bin] in these green panels have been scaled down by a factor of 10 for display reasons, as the aversive stimulus generates a highly time-locked response in 100% of the presentations, thus creating much higher event counts in the respective time bin). The nontarget presentations (± 1, ± 2, ± 3, ± 4) have not been collapsed to one histogram, but have been ordered in four different rows in the graph with respect to their perceptual 'distance' to the target, i.e. frequency distance in the auditory session, and spatial distance on the piano keyboard in the motor session, respectively. The category "± 1" designates the neighboring key on the keyboard (to the left and right, respectively). The maximum distance to the target withing the 5-tone-space is ± 4. Non-musicians (NM) are depicted in the upper 5 rows and professional pianists (PP) in the lower five rows. t = 0 (dotted line) refers to tone (CS) onset or keystroke, respectively. The dashed line marks the onset of the airpuff (US, at t = 200 ms). Note that only during the Auditory Conditioning and only for the target tone the US was present (Green panels). Please note the presence of the two twitch-related peaks during Session 2 in the Pianist group.

Figure 2 gives an overview of an entire dataset for the 200 ms group (the panels for the other CS-US delays are similar). As the PSTH display reveals, the two peaks of the response during the conditioning in Fig. 1 can be described by two classes of events. The first response component (the time series' small peak 100 ms after the CS in Fig. 1, T1) is generated almost exclusively by twitches of small amplitude but precise time locking (Fig. 2, darker colors). The second component of the response (B_US_1) is generated mainly by full closures of the eyelid (Fig. 2, lighter colors). The ramp in the time averages is missing in the PSTH display because only the incidences of peak events (local maxima of the time signal) are included. Therefore, any slow slope of the eyelid does not appear in a PSTH.

#### Session 0: baseline condition

The left column in Fig. 2 shows the spontaneous eyeblink distribution in the subjects before the conditioning procedure, but with the auditory tones already being played. The differentiation target vs. nontarget does not apply in this session because it is not until Session 1 that the target tone is experimentally distinguished. Eyeblinks are distributed evenly and are statistically not locked to the onset of the piano tones, and do not differ between the groups (two-tailed t-test, t = 1.37, p > 0.05). In the nonmusician group, however, one can detect an increased likelihood of small amplitude eye twitches related to the tone. This can be related to an auditory startling effect, and interestingly this is missing in the professional pianist group, most likely due to the high degree of familiarity with the specific sound.

#### Session 1: auditory conditioning

The most salient difference in the data for this session (Fig. 2, middle column) is of course the eyeblink immediately triggered by an actual airpuff to the eye in the target condition (Fig. 2, green panels). In addition to this, the following differences to baseline can be observed: (1) Following the tone presentation, but preceding the airpuff, the incidence of small eye twitches is increased in both groups. This is particularly interesting in the pianist group, as they did not show a startle reaction to the tones under baseline circumstances, but with the occasional coupling of a piano sound and an aversive stimulus, now the piano tones (including 'harmless' nontargets) evoke a startle reaction. Additionally, in the pianist group there seems to be a correlation of eyeblinks (light blue/light green) with the perceptual distance of stimulus frequency and conditioned frequency, however, this correlation is not significant (see below).

#### Session 2: silent tapping

In this session (Fig. 2, right column), the behavior of the non-musicians is back to the distribution observed at baseline. Neither eyeblinks nor twitches are related to the active keypress event at all.

In the pianists, however, a different pattern can be seen. In addition to an overall increased spontaneous blink rate, two distinct twitch responses are event-related to the pressing of a key: One twitch occurs exactly at the moment of the motor execution itself (t = 0). The second twitch occurs at the time point at which during the auditory session an airpuff was expected. This is a crucial observation because the twitch is timelocked to a silent motor event in Session 2, but the airpuff was timelocked to an auditory event in Session 1, suggesting feature similarities between tone and keypress within the stimulus representation in the pianist group. Please note from Fig. 2 that the two twitch peaks are present for both target-related and nontarget-related keystrokes, i.e. no target-specificity can be observed.

### Signal-response analysis

Based on the spike data that had been prepared for the PSTH graphs, we performed analyses of various SDT measures (Signal Detection Theory, see Methods) defining either blinks or twitches as the detector response.

#### Overall excitability

**Figure 3:**
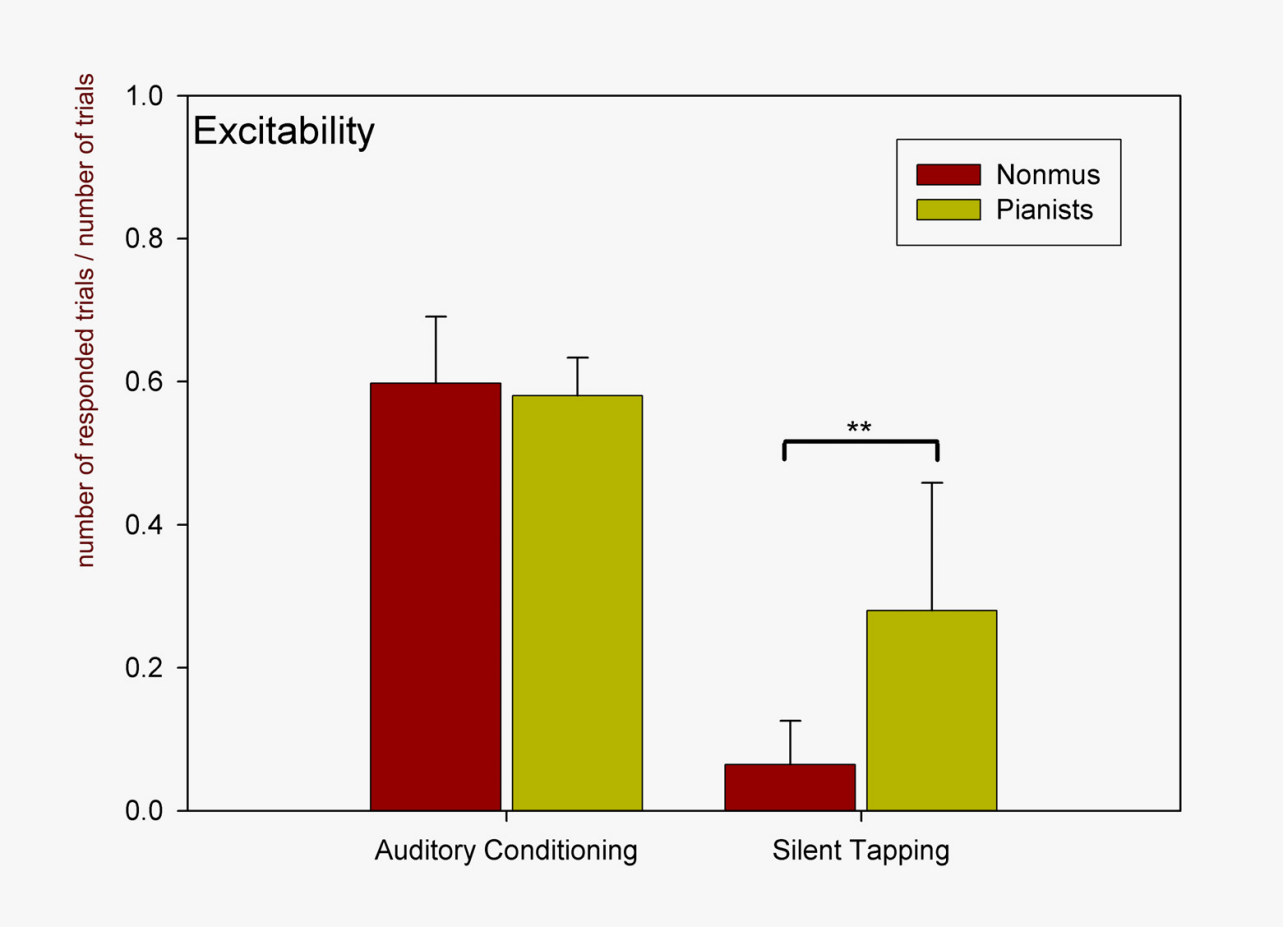
**Overall Excitability**. Excitability (ratio of the number of trials containing a positive response to the total number of trials) of eyeblink events in the two experimental conditions. Red: NM group; Yellow: PP group.

To test for the overall excitability of the subjects' eyeblink (response to any stimulus type), the ratio of the number of trials containing a positive response to the total number of trials was calculated (Fig. 3). Excitability measures all responses, regardless of whether they are Hits or False Alarms, respectively, to account for the subjects' general inclination to respond to any auditory stimulus or motor action with an eyeblink. Unlike the Auditory Condition, where no significant group difference was found, the motor condition (Silent Tapping) revealed a highly significant difference between the two groups (p < 0.01, df = 1, F = 8.478). While the number of responses dropped to a low level in the NM in this condition (during which no sound is heard), the professional pianists blinked more often.

#### Sensitivity

Although the overall excitability of an eyeblink can be increased by the conditioning procedure, so that in the pianists the effect is carried over to a motor task, the PSTHs in Fig. 2 suggest that this effect is not specific to the target stimulus.

#### Blinks

In the pianist group, the blinks (Fig. 2, light blue) show a tendency (n.s.) to occur not only for the target tone but also for nearby tones, the likely reason for which is a processing interference due to perceptual frequency discrimination thresholds. This explanation is supported by the observation that this tendency is present only for the shortest processing time (200 ms CS-US delay).

#### Twitches

The twitches (Fig. 2, dark blue), despite the strong overall effect (as they are present in non-musicians in Session 1 only, but in pianist in both Sessions 1 and 2), show no specificity for the target frequency.

**Figure 4:**
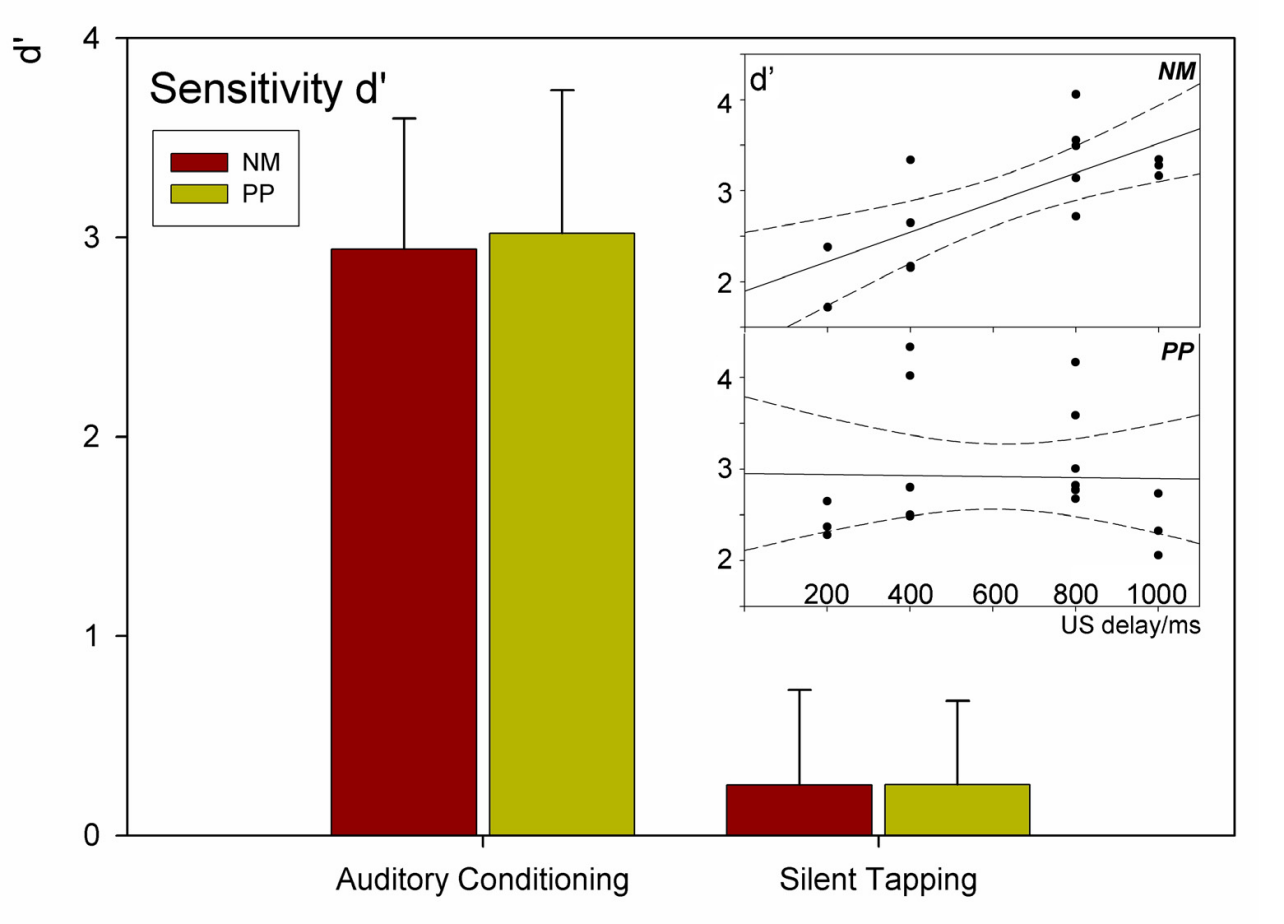
**Sensitivity**. Eyeblink Sensitivity d' for the two groups in Sessions 1 and 2. d' was high in the conditioning session due to the presence of the US. In session 2, d' drops to a small value indicating no specificity for the key related to the target tone. In any part of the experiment, no sensitivity difference between the groups is observed. NB: The graph shows eyeblink sensitivity only. Twitches, however, display an equally low d' in both Sessions 1 and 2. Red: NM group; Yellow: PP group. Inset: Correlation of d' with the US-CS delay in Session 1. A positive correlation is present in the non-musician group (upper panel, r = 0.8, p < 0.05) but not in the musician group (lower panel, r = 0.4, p = n.s.). In Session 2 (not shown), no positive correlation is found in either group.

The sensitivity d' (Fig. 4) incorporates both the Hit rate and the False Alarm rate, in order to determine if a signal detector is capable not only of detecting a stimulus in the presence of targets and nontargets, but also of discriminating the target stimulus^1^. d' does not differ between the two groups (Fig. 4) in either of the sessions. A more detailed analysis showed that the groups differed in their z-scores for Hits (p < 0.01) as well as in their z-scores for False Alarms (p < 0.01) (the PP group having the higher values in both cases). Since both z-scores contribute to the equation for d', they effectively cancel each other out, resulting in a similar sensitivity value for the two groups.

Conclusively, this means that in the Silent Tapping session the non-musicians tend to have a large number of Misses together with a large number of Correct Rejections, while the pianists have a large number of Hits together with a large number of False Alarms. In other words, the NM group hardly ever blinks or twitches at all while silently pressing keys, whereas the PP group blinks (and mostly twitches) very often, regardless of whether the key belongs to the target or not.

This is in essence the same result as in the excitability analysis (Fig. 3), indicating that the PP group does (unspecifically) associate keystrokes with the auditory CS, while the NM group does not.

To determine whether or not longer CS-US delays may facilitate the capability to process the pitch of the stimulus, Fig. 4 (inset) shows how d' depends on the CS-US interval in the Auditory Conditioning procedure. Pianists have an equal distribution of d' for all tested delays, although the subjects' data are widely scattered across the range, thus not allowing for a consistent interpretation. The non-musicians seem to benefit slightly when they are granted a longer stimulus processing time after the CS, as their sensitivity is poor for very short delays (200 ms). For Session 2, no such correlation could be found (because in the non-musicians the event-related blink/twitch activity was abolished altogether, as demonstrated in Fig. 2).

#### Performance in the distractor task

Although the stroop task itself provides data that are only secondary for the question at hand, it nevertheless is important to check whether or not the two subject groups performed equally well in this distractor condition. Otherwise the possibility of a cross-interference affecting the primary 'task' (the conditioning) could not be ruled out. The two groups performed at equal sensitivity (Nonmusicians 0.62 ± 0.09, Pianists 0.64 ± 0.09, p = 0.56), specificity (Nonmusicians 0.75 ± 0.04, Pianists 0.77 ± 0.03, p = 0.27), and efficiency (Nonmusicians 0.72 ± 0.04, Pianists 0.73 ± 0.03, p = 0.29) for detecting the target stroop color.

#### Awareness of the US

A final question to be addressed is the degree of awareness of the fact that one particular target tone was associated with the aversive stimulus. If, despite the attention-consuming distractor task, the subjects had managed to learn which tone was connected with the airpuff, one could expect that during the Silent Tapping task, particularly the pianists might tend to have avoided pressing the respective piano key that is suspected to trigger further airpuffs. However, a t-test showed that both groups, when being free to press the keys voluntarily, did not show a bias towards omitting the target key in favor of the nontarget keys.

## Discussion

This study presents data suggesting different behavioral reactions to a motor task in pianists and non-musicians following an auditory classical conditioning training of the eyeblink reflex.

The classical conditioning procedure of the eyeblink reflex yielded a typical aversive anticipatory reaction, consisting of a short-latency twitch-like lid movement and a long-latency ramp-like movement.

The findings clearly demonstrate a carry-over effect of the conditioned response into the motor domain in trained pianist, thus providing evidence for the main hypothesis of the experiment.

Testing for the additional hypotheses 1 and 2 (effect-specificity for timing and frequency), provided a negative result, suggesting that the main effect is group-specific but not parameter-specific.

### Absence of frequency-specificity

Significant frequency discrimination was not found throughout the subjects. While detection of a sound is possible without auditory cortex, discrimination is not [11,12]. In other words, discrimination is a much more complex task than detection and involves additional structures to those involved in detection. Additional processing demands in the present study included the four CS- stimuli which were employed instead of one (unlike most trace eyeblink conditioning studies[13]), and the fact that the CS- were very similar to the CS+. The discrimination of a note E, for example, between the nearby higher pitches of F, G, as well as lower pitches of C, D, is probably much more difficult a task than, for example, the discrimination of a pure tone against white noise in a typical oddball paradigm. In both perceptual and temporal learning paradigms [e.g. 1,2], it takes hours and days, respectively, to establish a stable effect. This most likely can be attributed to the multitude of sensory inputs concurrently bombarding the system, of which the brain has to make sense by attaching markers to behaviorally relevant stimuli and deriving underlying temporal regularities. Especially the learning of short intervals takes thousands of trials, a number which in the current experimental setup could not be achieved.

### Transfer of the reflex response to the motor domain

The Silent Tapping session represents the core of the experimental design. It addresses the question of whether or not a motor action never coupled with an aversive stimulus, nonetheless leads to eye blinking simply because of prior association with a tone serving as a Pavlovian CS, on the basis of the specific sensorimotor experience in pianists.

The results indeed indicate a higher total likelihood of blinking during the silent tapping session in the pianist group. However, a consistent event timing could only be seen for twitches, not for full blinks, which makes it difficult to interpret these results in terms of a stimulus-triggered, classically conditioned response. Despite this lack in precise timing of the tapping-related eyeblinks, the fact that there is a significantly higher blink rate during tapping in pianists compared to non-pianists, illustrates that a transfer of the auditory conditioned eyeblink reflex to the tapping task occurs in the first group, but not the latter.

The possibility that pianists, in general, have an enhanced blink rate compared to the rest of the general population, had been taken into account for by testing the spontaneous reactions before the training session. Although it is not impossible that the training and the multiple exposure to corneal airpuffs could have resulted in higher blink rates due to the temporary irritation of the cornea, *there is no reason to think that pianists' eyes in general would be more irritable than non-musicians'*.

What exactly it is that is transferred from the Auditory Conditioning session to the Silent Tapping session, comes down to the question of whether the information about the reflex CS is being encoded by (and maybe stored in) auditory or motor areas of the brain, or both. The literature so far suggests that the involuntary co-activation can be evoked in both directions, auditory-to-motor [6], and motor-to-auditory [4,6]. The following scenarios are proposed:

#### (1) Motor co-activation during session 1

It can be argued that the strong audiomotor link in the pianist's brain already becomes active during the Auditory Conditioning session. This model suggests that during conditioning with piano tones, the familiar tones induce covert movement preparation. Therefore, the effect that becomes visible during the tapping task had already been mediated during the preceding conditioning round. This could account for the lack of target discrimination in the tapping part. Absolute Pitch (six of the PP subjects) has been considered as a confound, but t-tests of this subgroup against the rest of the pianists have not shown differences for any of the measured parameters. Although this interpretation is not impossible, the assumption that motor preparation takes place for single isolated tones without a melodic context (i.e., not embedded in a motor sequence) is not very plausible.

#### (2) Feedback anticipation during session 2

Conversely, it is conceivable that the pianists acquire normal reflex conditioning to the auditory cue. While silently depressing piano keys afterwards, the feedback anticipation of the expected tone leads to an increased blink rate. This viewpoint would indicate that maybe an auditory mental representation of the CS alone can be sufficient to evoke the memory trace of the US.

## Conclusion

This paper reports a phenomenon of involuntary reflex responses after classical conditioning to a sensory stimulus, elicited not by the stimulus itself, but by a voluntary motor action that is arbitrarily associated with the conditioning stimulus through long-term training. Whether the neuro-cognitive mechanism underlying this sensorimotor effect is coupled to the motor, or rather to the sensory, systems involved, cannot be conclusively disentangled by this initial experimental design. The most reasonable theory may be something in between a motor-centered and a sensory-centered argumentation – considering the underlying brain substrate not as a highly unidirectional cause-and-effect driven computational device, but rather as a distributed, branched, network structure, in which *association *is the fundamental processing principle. Thus, it might be reasonable to speak of common representations rather than of dissociated elements.

The exciting question of whether these two interpretations are just different hallmarks of a shared mechanism, or if there is a true dissociation that allows for experimental tests of one of the models, calls for further, more refined experiments utilizing cross-modal reflex conditioning paradigms. This could represent a promising approach in the field of expert sensorimotor behaviors.

## Methods

### Subjects

Two groups of subjects were recruited. The non-musician group (NM) consisted of 14 righthanded students [17] with no formal instrumental training (13 women; average age 22.2 ± 3.2 years). The professional pianist group (PP) consisted of 17 professional pianists and music students with piano as one of their principal instruments (13 women, average age 24.2 ± 5.6 years; age of commencement of instrumental training 6.2 ± 1.8 years with an accumulated lifetime practice of 17.9 ± 5.6 years; present practice schedule 23.0 ± 11.8 hours/week). Two of the PP group participants were left-handed. Six of the PP group participants reported Absolute Pitch. All subjects gave written informed consent before the beginning of the experiment.

Since the conditioning training could only be applied once to each participant, each subject was randomly assigned to be conditioned to one out of five possible target tones at one out of four possible CS-US latencies. The number of subjects within each factor level (combination of parameters) will be given below.

### Experimental setup

The participants sat on a comfortable chair with armrests in front of an electronic keyboard used in the last part of the experiment (silent tapping condition).

Auditory stimuli were FM-synthesized single piano tones with a typical attack-decay-envelope. The tones were allowed to sound (decaying) until the US was presented. The fundamental frequencies of the five different tones (c', d', e', f', g') were 1046.5 Hz, 1174.7 Hz, 1318.5 Hz, 1396.9 Hz, and 1568.0 Hz. These step widths were chosen so as to be easily discriminable both by experts and non-musicians familiar with western-style music [18].

The stimuli were D/A converted by a PC soundcard (Terratec EWS64 XL) and delivered to the subject with an active loudspeaker (Klein & Hummel) at a distance of 90 cm in front of the subject (approximately acoustic free field conditions). They were presented at an average sound pressure level of 73 dB(A). Inter-stimulus silence in the lab was 39.8 dB (A).

Visual stimuli were presented on a computer screen at a distance of 90 cm in front of the subject.

For the delivery of the airpuffs and the measurement of the eyelid movements, we modified a noncontact technique for upper eyelid movements employed by [19] in squirrel monkeys. Using a computer-controlled magnetic valve, the airpuff (20 ms, 1–2 bar) was delivered to the left eye through a goggle-mounted nozzle. The eye-to-nozzle distance measured approx. 10 mm.

Eyelid movements were registered with the aid of a phototransistor (OP505) and an infrared light-emitting diode (OP165) close to the airpuff nozzle. Stimulus presentation and airpuff delivery were controlled using custom-made software.

The nozzle, the photo diode and the photo transistor were integrated into the plastic goggles so that the subject could comfortably look at the screen where the visual stimuli were delivered, and the subject's head did not have to be fixated relative to the nozzle/infrared device.

Closing of the eye changed the reflection of the emitted light and thus caused a change in the phototransistor signal. The signal was preamplified and fed into one channel of a modified 32-channel SynAmps™ amplifier and was recorded by means of NeuroScan™ software (sampling rate 200 s^-1^, low pass filter = 40 Hz, 24 dB/octave). During the silent tapping condition (see below), motor responses on the piano keyboard were coded into MIDI signals (Musical Instrument Digital Interface) and coregistered with the phototransistor data in the NeuroScan file.

### Experimental procedure

**Figure 5:**
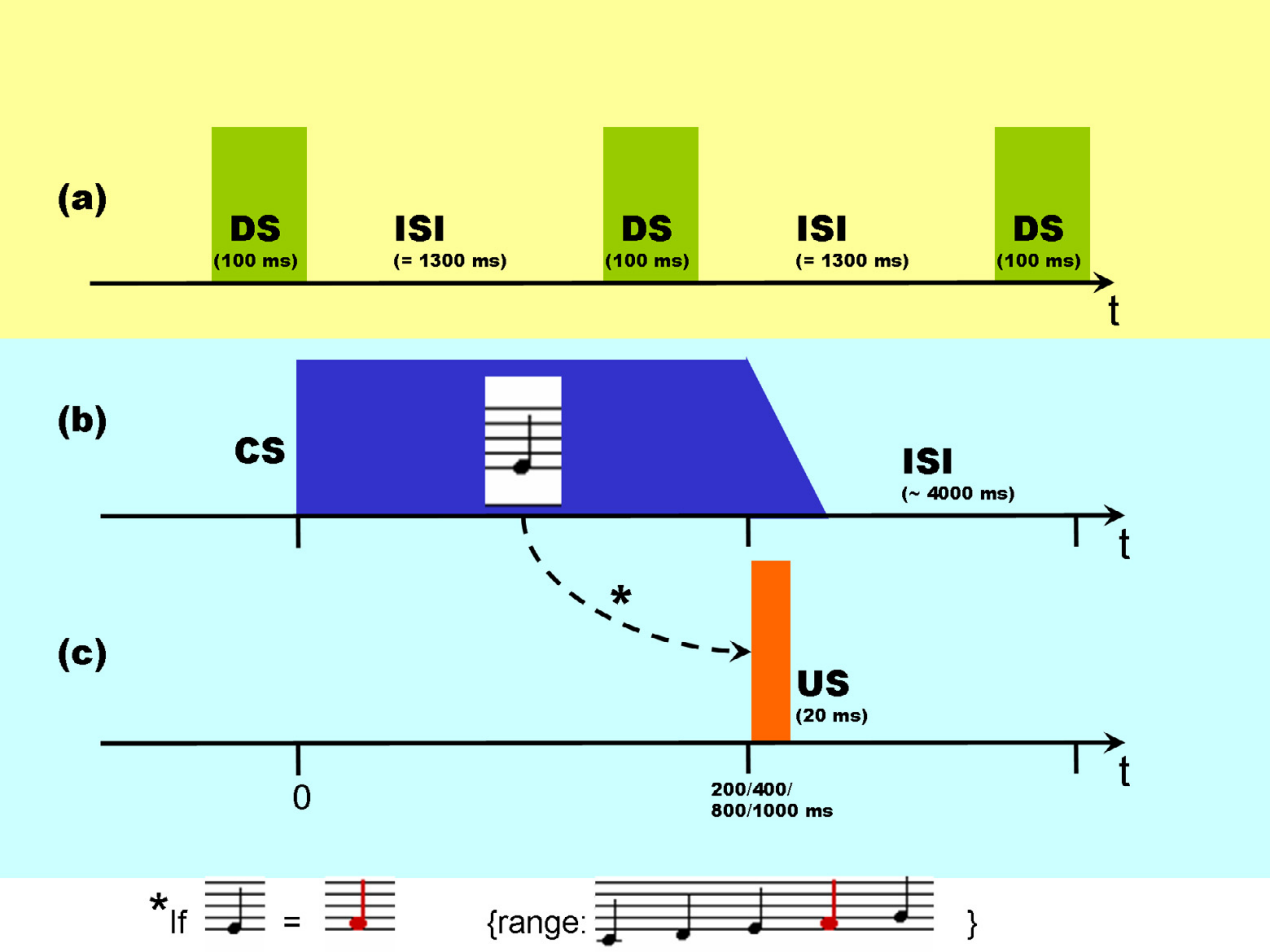
**Experimental paradigm for the Auditory Conditioning (Session 1)**. (a) Distractor task. To distract the subjects' attention away from the auditory stimuli, a visual Stroop task with alternating attention shift conditions was presented simultaneously but not phase-locked to auditory stimulation. The visual distractor stimulus (DS) is delivered with an ISI of 1300 ms. (b) The conditioned stimulus (CS) was one of the set of five possible piano tones (range indicated at bottom of figure) and was delivered at an ISI of 4000 ± 1500 ms (randomized). The musical notation is depicted in treble clef one octave lower than the actual stimuli, for display reasons. (c) The unconditioned stimulus (US) was delivered at the subject's eye whenever the particular tone (= target; = CS+ in (b)) sounded, but not for the remaining non-target tones. At the bottom of the figure, the note f is marked red as an example. NB: As indicated by different background colors, timeline (a) runs at the same time but independently from (b) and (c), i.e. (a) is not phaselocked to (b) and (c), while the latter two are phaselocked to one another at a preselected CS-US delay. Session 0 differs from the depicted paradigm in that no US is delivered (i.e. row (c) never happens); Session 2 differs in that a keypress is substituted for the auditory note onset in row (b), and row (c) is omitted (no actual US anymore).

The main experiment consisted of two different, but similarly designed sessions and a baseline condition. All three sessions were 'masked' from the subjects' attention by a demanding distractor task (see below):

• Session 0 (Baseline): Prior to the conditioning about 70 random stimulus presentations out of the set of five tones used during the Auditory Conditioning (see below) were performed to evaluate the eyeblink baseline under conditions identical to Session 1, but without the US (Fig. 5). This baseline served as an evaluation of spontaneous eyeblink rates, and of possible startle reflex blinking in response to the auditory stimuli.

• Session 1 (Auditory Conditioning) was the classical conditioning procedure (Fig. 5). Subjects were acoustically stimulated with 100 presentations of the randomized tones (ISI: 4000 ± 1500 ms). One of the tones (balanced between subjects and across groups) was assigned the conditioned stimulus (CS+). The unconditioned stimulus (US) was delivered only after the CS+ tone.

• Session 2 (Silent Tapping) required the subjects to place their right hands over the five piano keys corresponding to the five notes used in Session 1 (each finger over one key), and to voluntarily press down one of the keys about every 4–5 seconds. Equal distribution of the five fingers and of the inter-onset intervals (IOI) was monitored. The crucial manipulation was that the sound of the electronic piano was turned off, so that in this session neither the US nor the CS were present. The session was terminated after ~100 keystroke events.

In order to parametrically investigate the influence of the CS-US delay on the success of the conditioning procedure, four different delays were used during the study and randomly assigned to the participants, matched across the two subject groups (the number of subjects appear in brackets): 200 ms (n = 5), 400 ms (n = 9), 800 ms (n = 11), 1000 ms (n = 6).

Similarly, to investigate the influence of the relative position of the target frequency among the nontargets, for each of the subjects one of the five notes (c', d', e', f', g') was randomly chosen to represent the target stimulus (CS+) during the conditioning procedure, while the remaining four notes were the nontargets (CS-). This distribution was also matched across the two subject groups (the number of subjects appear in brackets): c'(n = 7), d'(n = 5), e'(n = 8), f'(n = 4), g' (n = 7).

To make sure that the classical conditioning effects occurred on an unintentional cognitive level, each part of the measurement was paralleled by an attentionally demanding distractor task, which the subjects were told to be the actual task of interest (Fig. 5). During this modified stroop task, the German words for RED, YELLOW, BLUE, WHITE were presented randomly on the screen for a duration of 100 ms and with an ISI = 1300 ms. The letters of the words were colored red, yellow, blue or white (luminance-matched) in a randomized sequence. The subjects were instructed to count every instance of, e.g., "red" during a session, but, depending on the background color of the screen (dark gray or black), to attend either to the actual color, or the semantic content of the words, respectively. The attention shift was prompted every 60 seconds. After the session, the subjects were asked to report their count, to verify whether they had been fully attending the distractor task during the whole session.

### Data analysis

Raw data were stored on a hard disk and converted to epoched NeuroScan file format. The epochs comprised the time window [-500 ms; + 1500 ms], with t = 0 (for the following event-locked analyses) at the onset of the CS in the auditory conditioning session, and at the keystroke event in the silent tapping session respectively. A total of 8389 event-related epochs were included in the analysis (3692 for the NM group, 4697 for the PP group). Subsequent analysis steps were performed with custom-made software.

### Averaging

Averaging of blink reflex responses can be beneficial in detecting very small signals which otherwise might be lost in the noise [20]. As the magnitude of the phototransistor signal largely depends on the position and angle in front of the eye, as well as on the anatomical and physiognomic geometry of the individual upper eyelids, each subject's data was normalized with regard to the maximum of the average individual eyeblink amplitude as response to the US in Session 1. The normalized data was then group-averaged.

### PSTHs

Peri-Stimulus-Time-Histograms (PSTH) were obtained from the epoched data by manually detecting peaks in the time-series. As the average data (cf. Results) had suggested that, in addition to the full eyelid-closure (in the following referred to as *blinks*), there seemed to be event-related responses of a smaller amplitude (*twitches*), which could not be reliably extracted by means of an automated peak-detection algorithm. Therefore, a blinded (with regard to the factor levels *subject, group *and *target tone*) observer extracted the events. The criterion for a twitch was any transient peak of amplitude less than 25% of the average amplitude of a blink in the same subject.

### Signal-response analyses

For an analysis of the subjects' responses we considered any eyeblink event between CS onset and the end of the epoch as a positive response. In the silent tapping session, events within a 200 ms window *before *the keystroke were equally considered a positive response as it was unclear whether an anticipatory effect could emerge during motor preparation. From the Signal Detection Theory (SDT) measures Hit (the presentation of a target tone yielded a CR), Miss (a target tone was not followed by the CR), False Alarm (a non-target tone induced an eyeblink), and Correct Rejection (a non-target tone not followed by an eyeblink), the Sensitivity d' was computed using standard expressions [21]. Additionally, we investigated a measure termed 'Excitability', which represents the number of responses per number of trials, regardless of whether the responses were Hits or False Alarms. Excitability therefore counts blinks in response to a CS irrespective of being a CS+ or CS-, and relates them to the number of trials thus providing for the inclination of the two groups to perform event-related blinks. For the Silent Tapping session (Session 2, 'Test'), during which the subjects could choose to play each of the five piano keys voluntarily, the relative distribution of the five keys, i.e., the Target vs. Nontarget ratio was calculated, where a ratio of one denotes a non-existing bias towards avoiding the silent key representing the individual target tone.

### Statistical analysis

The results of the signal-response analyses were subjected to a repeated measures MANOVA with between-subject factors *Musicianship *(i.e. group), *CS+ *(note that yielded a US in the training session), and *Delay *(delay between CS and US). The significance threshold was fixed at 0.05 (and 0.01 for highly significant).

## Abbreviations

CR, conditioned response

CS, conditioned stimulus

CS+, conditioned stimulus coupled with US

CS-, conditioned stimulus not coupled with US

IOI, Inter-onset interval

ISI, Inter-stimulus interval

NN, Non-musicians

PP, Professional Pianists

PSTH, Peri-Stimulus Time Histogram

SDT, Signal Detection Theory

US, unconditioned stimulus

## Authors' contributions

MB designed the experimental paradigm, conducted the experiments and analysis, and drafted the manusicript. UH built the experimental setup, performed the preprocessing of the data, and contributed to the manuscript. UJ and EA contributed to the hypothesis, design, results, discussion, and drafting of the manuscript.

## Note

During the Auditory Conditioning session, the sensitivity is naturally relatively high because of the airpuff, especially when the conditioned response does not develop to a full blink prior to the US but rather a ramp only (this is a methodological effect as ramp events are not included as response events for the PSTH analysis).
